# Impact of stringent non-pharmaceutical interventions applied during the second and third COVID-19 epidemic waves in Portugal, 9 November 2020 to 10 February 2021: an ecological study

**DOI:** 10.2807/1560-7917.ES.2022.27.23.2100497

**Published:** 2022-06-09

**Authors:** Ana Rita Torres, Ana Paula Rodrigues, Mafalda Sousa-Uva, Irina Kislaya, Susana Silva, Liliana Antunes, Carlos Dias, Baltazar Nunes

**Affiliations:** 1 Department of Epidemiology, National Health Institute Doutor Ricardo Jorge, Lisbon, Portugal; 2 Public Health Research Center, NOVA National School of Public Health, Lisbon, Portugal; 3 Comprehensive Health Research Center (CHRC), Lisbon, Portugal; 4 Centre of Statistics and its Applications, Faculty of Sciences, University of Lisbon, Portugal

**Keywords:** COVID-19, NPI, surveillance

## Abstract

**Background:**

Non-pharmaceutical interventions (NPIs) were implemented worldwide to control the spread of SARS-CoV-2.

**Aim:**

To evaluate the impact of tiered NPIs and a nationwide lockdown on reduction of COVID-19 incidence during the second and third epidemic waves in Portugal.

**Methods:**

Surveillance data on laboratory-confirmed COVID-19 cases were used to conduct an interrupted time series analysis to estimate changes in daily incidence during a second wave tiered NPI period (9 November–18 December 2020), and a third wave lockdown period without (15–21 January 2021) and with school closure (22 January–10 February 2021).

**Results:**

Significant changes in trends were observed for the overall incidence rate; declining trends were observed for tiered NPIs (−1.9% per day; incidence rate ratio (IRR): 0.981; 95% confidence interval (CI): 0.973–0.989) and a lockdown period without (−3.4% per day; IRR: 0.966; 95% CI: 0.935–0.998) and with school closure (−10.3% per day, IRR: 0.897; 95% CI: 0.846–0.951). Absolute effects associated with tiered NPIs and a lockdown on a subsequent 14-day period yielded 137 cases and 437 cases per 100,000 population potentially averted, respectively.

**Conclusion:**

Our results indicate that tiered NPIs implemented during the second wave caused a decline in COVID-19 incidence, although modest. Moreover, a third wave lockdown without school closure was effective in reducing COVID-19 incidence, but the addition of school closure provided the strongest effect. These findings emphasise the importance of early and assertive decision-making to control the pandemic.

## Introduction

Between January 2020 and March 2021, more than 90 countries worldwide implemented different levels of restrictions to slow the transmission of severe acute respiratory syndrome coronavirus 2 (SARS-CoV-2) and mitigate the severe impact of coronavirus disease (COVID-19) on morbidity and mortality [1].

During the first epidemic wave, Portugal enforced a nationwide lockdown from 16 March to 3 May 2020 as well as a combination of non-pharmaceutical interventions (NPIs). Such NPIs included a stay-at-home mandate, closure of schools and all non-essential businesses, individual movement restrictions for non-essential activities, closing of international borders for non-residents, banning of mass gatherings, visitation restrictions in long-term care facilities and teleworking [2]. In the second epidemic wave (October–December 2020), to avoid such stringent constraints on social life and economy while controlling the health crisis and following World Health Organization (WHO) recommendations, a targeted intervention was established in Portugal from 9 November 2020 onwards [3]. Municipalities were classified according to their registered incidence of COVID-19 cases every 2 weeks, using a four-tiered approach, and NPIs with different levels of stringency were applied. Along with the mandatory use of face masks in indoor and outdoor spaces and a nationwide curfew from 23.00 to 5.00 in mainland Portugal, municipalities placed into tiers with more stringent NPIs had weekend lockdowns implemented [4]. However, during 24–26 December 2020, these measures were lifted across Portugal to allow for family reunions on Christmas Day.

Nevertheless, with the dramatic increase of COVID-19 cases in the third epidemic wave (late December 2020–March 2021), in which Portugal had reached a 14-day incidence rate of 1,667 cases per 100,000 population on 29 January, more strict measures were issued in order to reduce the impact on the population and health services [5]. On 15 January 2021, a nationwide lockdown was set. Restrictions were similar to those experienced during the first epidemic wave lockdown, but schools were allowed to remain open. Seven days later (on 22 January), in order to curb the dramatic increase of hospitalisations and admissions to intensive care units (ICU), approaching saturation of the healthcare system, schools were also closed until 12 March 2021.

The implementation of stringent NPIs helped control the COVID-19 pandemic in several countries [6,7]. In Portugal, findings suggest that a nationwide lockdown during the first epidemic wave helped to reduce the number of COVID-19 cases, hospitalisations and deaths [8]. The aim of this work was to evaluate the impact of tiered NPIs and nationwide lockdown applied in Portugal during the second and third epidemic waves in reducing the incidence of COVID-19. More specifically, we evaluate COVID-19 cases pre- and post-restrictive measures for each of the five mainland regional health administrations.

## Methods

### Study design and population

We developed an ecological study using an interrupted time series analysis. This methodology analyses the longitudinal effects of an intervention on a given outcome, comparing an expected trend without intervention to the trend found after the intervention [9].

Our study population comprised residents in mainland Portugal. We excluded Madeira and Azores islands from the analysis, as these autonomous regions applied independent NPIs to control COVID-19 spread, since the beginning of the pandemic.

### Outcome variables

Surveillance data on daily COVID-19 laboratory-confirmed cases – from across Portugal overall and in the five mainland regional health administrations (North, Center, Lisbon and Tagus Valley, Alentejo and Algarve) – were made available by the Directorate General of Health (DGS) in Portugal [10]. The provided dataset also includes the date of symptom onset, date of diagnosis and date of confirmation/notification for each case.

To account for the missing data on symptom onset (ca 63%), multiple imputation based on the empirical distribution of the time between onset and diagnosis was carried out to produce 100 datasets. The empirical distribution of the time between onset to diagnosis was stratified by region, age group and day of the week and computed on the last 15 days (from the date of diagnosis) for each case with missing data to account for changes in time. The daily incidence, used to perform all statistical analysis, was given by the median number of cases by date of symptom onset in all imputed datasets [11].

The most recent resident population estimates, extracted from Statistics Portugal, were used as denominators to compute incidence rates [12].

### Covariates

Mean daily temperatures used to analyse a possible existence of a confounder bias were made available by the Portuguese Institute for Sea and Atmosphere (IPMA; https://www.ipma.pt). We associated Portugal and each mainland region with an average temperature as follows: for Portugal, we used an average of the daily mean temperatures of the mainland meteorological stations (n = 98) located across the country whereas, for each region, average temperature is tabulated only for its capital city.

### Study period

The observation period for our analysis was chosen considering several factors: (i) a sufficient (≥ 12 days) and equal timeframe before and after each restrictive measure implementation [13], (ii) controlling for effects of simultaneously occurring interventions [13] (iii) and the onset of the second and third pandemic waves [11].

**Figure 1:**
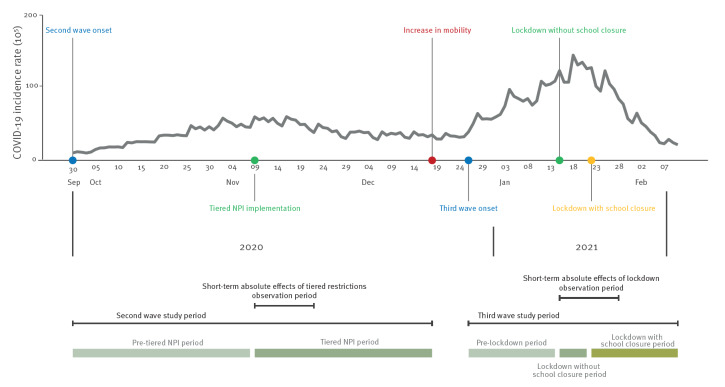
COVID-19 incidence rates^a^ and timeline of tiered non-pharmaceutical interventions and lockdown implementation, Portugal, 30 September 2020–10 February 2021

COVID-19: coronavirus disease; NPI: non-pharmaceutical interventions.

^a^ Per 100,000 population.

To assess COVID-19 counts by symptom onset in Portugal before and after tiered NPIs implementation (second wave study period), we compared two periods (Figure 1): (i) the pre-intervention period starting in 30 September 2020 (considering the onset of the COVID-19 second pandemic wave) until 8 November 2020; (ii) tiered NPIs period from 9 November 2020 through 18 December 2020 (considering a sharp increase in mobility that occurred after the beginning of Christmas holidays, especially because of retail trade) [14].

Time intervals regarding study periods for the second and third COVID-19 epidemic waves and short-term absolute effects observation periods are depicted in black.

During tiered NPIs period, municipalities were classified into four tiers (Tier 1–Tier 4), although NPIs implemented did not differ between Tiers 1 and 2 and between Tiers 3 and 4. NPIs were more stringent for municipalities placed into Tiers 3 and 4, which included weekend lockdowns, compared to municipalities placed into Tiers 1 and 2 [4].

To assess COVID-19 counts by symptom onset in Portugal before and after lockdown implementation (third wave study period), we considered three periods (Figure 1): (i) the pre-lockdown period from 26 December 2020 (considering the increase in COVID-19 cases after Christmas [11]) through 14 January 2021, (ii) the lockdown without school closure period from 15 through 21 January 2021, (iii) the lockdown with school closure period from 22 January through 10 February 2021. During the pre-lockdown period, tiered NPIs similar to the ones implemented during the second epidemic wave were set, with the addition of a weekend lockdown after New Year’s Eve, across all mainland Portugal.

### Statistical analysis

We conducted an interrupted time series analysis to estimate changes in COVID-19 daily incidence rates before and after tiered NPIs and before and after lockdown implementation. Separate models were fitted to assess tiered NPIs and lockdown implementation.

In infectious diseases, dependence among neighbouring observations in a time series is often present because the number of newly infected individuals at a defined moment depends on the number of previously infected individuals in the population (‘true contagion’) [15]. Therefore, for each regional health administration, we fitted a negative binomial regression model to account for overdispersion due to autocorrelation, with daily COVID-19 incident cases as the primary outcome of interest. When needed, we also included a first-order autoregressive term (lagged residuals) to account for residuals autocorrelation [16].

Explanatory factors for tiered NPIs included the linear effect of time (slope) and change in trend after start of tiered NPIs (change in slope). Explanatory factors for the lockdown included the linear effect of time (slope), change in trend after start of lockdown without closure of schools (change in slope) and change in trend after school closure (change in slope). Population in each region was included in all models as an offset variable.

Due to SARS-CoV-2 median incubation period of 5 days, we included a lag of 5 days in variables regarding each intervention [17].

The statistical models used in our study, for the tiered NPIs and lockdown, were given as follows:


$$(tiered\;NPIs)\;logE\left(Y_{t}\right)=\beta_{0}+\beta_{1}{Time}_{t}+\beta_{2}{Time}_{t}{Lock2}_{t-5}+log\left({Pop}_{t}\right)$$

$$(lockdown)\;logE\left(Y_{t}\right)=\alpha_{0}+\alpha_{1}{Time}_{t}+\alpha_{2}{Time}_{t}{Lock3}_{t-5}+\alpha_{3}{Time}_{t}{School}_{t-5}+log\left({Pop}_{t}\right)$$

where *Y_t_* is a daily count of COVID-19 cases by time of symptom onset (t); *β_0_* and *α_0_* are baseline incidence rates; *Time_t_* is time in days since the start of the analysis observation period; *Lock2_t-5_*, *Lock3_t-5_* and *School_t--5_* are dummy variables representing, respectively, tiered NPIs, lockdown and school closure (0 in pre-intervention period, 1 otherwise); *β_1_* and *α_1_* are slopes of the outcome variable until tiered NPIs and lockdown implementation; *β_2_*, *α_2_* and *α_3_* are changes in slope after, respectively, tiered NPIs, lockdown without school closure and lockdown with school closure implementation; *Pop_t_* represents the population at time *t*.

Daily percentage change of COVID-19 incidence in pre and post-restrictive periods was calculated as 100% (IRR (incidence rate ratio)−1), where IRR for tiered NPIs and lockdown without and with school closure, respectively, was estimated as exp(β_1_ + β_2_), exp(α_1_ + α_2_) and exp(α_1_ + α_2_ + α_3_). The short-term absolute effects of tiered NPIs and lockdown was obtained by subtracting the predicted number of COVID-19 cases for each intervention period from the expected number of COVID-19 cases in the absence of interventions, considering a 14-day period for comparability purposes (9–22 November 2020 for tiered NPIs and 15–28 January 2021 for lockdown) (Figure 1).

The level of significance was set at 5% for all tests. Goodness-of-fit of the models was assessed by graphical analyses of residuals and Pearson’s Chi-squared test.

All statistical analyses were performed using R version 3.5.1 [18]. For interrupted time series analysis, we used the ‘MASS’ package and for residual diagnostics, we used ‘DHARMA’ package.

### Adjusting for confounding

The third COVID-19 epidemic wave in Portugal was coincident with a cold spell in January 2021, which was the fourth-coldest month on record in 20 years [19]. Therefore, daily average temperature was introduced in our regression models to control for potential meteorological confounding, as follows:


$$(tiered\;NPIs)\;logE\left(Y_{t}\right)=\beta_{0}+\beta_{1}{Time}_{t}+\beta_{2}{Time}_{t}{Lock2}_{t-5}+\beta_{3}{Temp}_{t-5}+log\left({Pop}_{t}\right)$$

$$(lockdown)\;logE\left(Y_{t}\right)=\alpha_{0}+\alpha_{1}{Time}_{t}+\alpha_{2}{Time}_{t}{Lock3}_{t-5}+\alpha_{3}{Time}_{t}{School}_{t-5}+\alpha_{4}{Temp}_{t-5}+log\left({Pop}_{t}\right)$$

where *Temp_t-5_* represents average daily temperature with a lag of 5 days, consistent with COVID-19 median incubation period.

### Sensitivity analyses around the lag period

Because COVID-19 incubation period estimates are based on emerging evidence, we examined the robustness of our findings if the lag period used in our regression models, regarding variables representing restrictive measures, varied between the 5^th^ and 95^th^ percentiles estimated for the median incubation period (2–12 days) [17].

## Results

### COVID-19 incidence rate

At the onset of the second wave, an increasing trend in COVID-19 incidence was observed in Portugal for the pre-tiered NPIs study period, peaking around tiered NPIs implementation (58.8 daily cases per 100,000 population, on 16 November 2020) (Figure 1 and Supplementary Figure S1: COVID-19 incidence and average temperature). Table 1 shows the proportion of Portuguese municipalities per region featuring stringent NPIs from 9 November to 18 December 2020. Considering the municipalities with at least one period (14 days) of more stringent NPIs, North and Lisbon and Tagus Valley were the regions with a higher proportion of municipalities under more restrictive measures. Additionally, North had a higher proportion of municipalities under more stringent measures during the entire examination period.

**Table 1:** Proportion of municipalities with stringent non-pharmaceutical interventions, stratified by regional health administration, Portugal, 9 November–18 December 2020 (n = 278 municipalities)

Regional health administration	Municipalities per region	Proportion of municipalities with stringent NPIs	
	n = 278	At least one 14-day period	Entire period
North	86	90.7	58.1
Center	100	64.0	7.0
Lisbon and Tagus Valley	18	100.0	16.7
Alentejo	58	44.8	3.4
Algarve	16	6.3	0.0

COVID-19: coronavirus disease; NPI: non-pharmaceutical interventions.

NPIs were considered as stringent if they included lockdowns as a measure of physical distancing.

At the onset of the third wave, an increasing trend was observed for pre-lockdown study period (26 December 2020–14 January 2021), peaking soon after the lockdown implementation without school closure (141.7 daily cases per 100,000 population on 18 January 2021) (Supplementary Figure S1: COVID-19 incidence and average temperature). During this period, a sharp decrease in daily average temperatures occurred, returning to its normal values for time of the year (8.8 °C) around school closure implementation. A few days after the lockdown implementation, a marked declining pattern in COVID-19 incidence trend was observed (Figure 1).

### Trends in COVID-19 incidence rate

**Figure 2:**
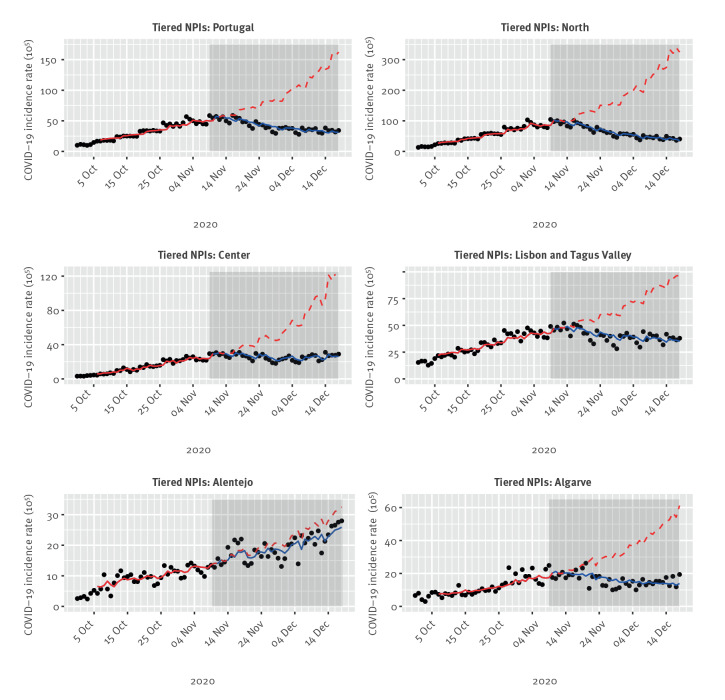
COVID-19 daily incidence rates^a^ before and after tiered non-pharmaceutical interventions implementation, across Portugal and in five regional health administrations, 30 September–18 December 2020 (n = 297,754)

COVID-19: coronavirus disease; NPI: non-pharmaceutical interventions.

^a^ Per 100,000 population.

In pre-tiered NPI study period (30 September–8 November 2020) after the second wave onset, the overall COVID-19 incidence rate increased in average by 2.8% per day (IRR: 1.028; 95% CI: 1.025–1.031). Significant increasing trends were observed in all regions, with the highest increase among Center (3.8% per day; IRR: 1.038; 95% CI: 1.035–1.042) and North (3.3% per day; IRR: 1.033; 95% CI: 1.030–1.035) (Table 2 and Figure 2).

**Table 2:** Daily trends in the COVID-19 incidence rate before and after tiered non-pharmaceutical interventions implementation, across Portugal and in five regional health administrations, 30 September–18 December 2020 (n = 297,754)

Area	Study period				Change in trend	95% CI	p value^a^
	Pre-tiered NPIs 30 Sep–8 Nov 2020		Tiered NPIs 9 Nov–18 Dec 2020				
	IRR	95% CI	IRR	95% CI			
Portugal	1.028	1.025–1.031	0.981	0.973–0.989	0.954	0.949–0.959	< 0.001
**Regional health administration**							
North	1.033	1.030–1.035	0.969	0.962–0.977	0.939	0.934–0.943	< 0.001
Center	1.038	1.035–1.042	0.993	0.983–1.002	0.956	0.950–0.962	< 0.001
Lisbon and Tagus Valley	1.019	1.017–1.022	0.990	0.983–0.998	0.971	0.967–0.976	< 0.001
Alentejo	1.021	1.017–1.025	1.014	1.002–1.026	0.993	0.986–1.001	0.085
Algarve	1.029	1.023–1.035	0.986	0.971–1.002	0.959	0.949–0.968	< 0.001

CI: confidence interval; COVID-19: coronavirus disease; NPI: non-pharmaceutical intervention; IRR: incidence rate ratio.

^a^ Two-sided Wald test p values were obtained from negative binomial regression models. A p value < 0.05 was considered evidence of statistical significance.

The shaded grey area represents the period after implementation of tiered NPIs. Red and blue solid lines represent the fitted incidence rates before and after tiered NPIs, respectively. The red dashed line represents the predicted incidence rate in a contrafactual scenario, i.e. if tiered NPIs had not been implemented.

In the tiered NPIs study period (9 November–18 December 2020), a statistically significant change in trend was observed for the overall incidence rate for 4/5 regions excluding Alentejo. Significant declining trends were observed in mainland Portugal (−1.9% per day; IRR: 0.981; 95% CI: 0.973–0.989), North (−3.1% per day; IRR: 0.969; 95% CI: 0.962–0.977) and Lisbon and Tagus Valley (−1.0% per day; IRR: 0.990; 95% CI: 0.983–0.998) (Table 2 and Figure 2).

**Figure 3:**
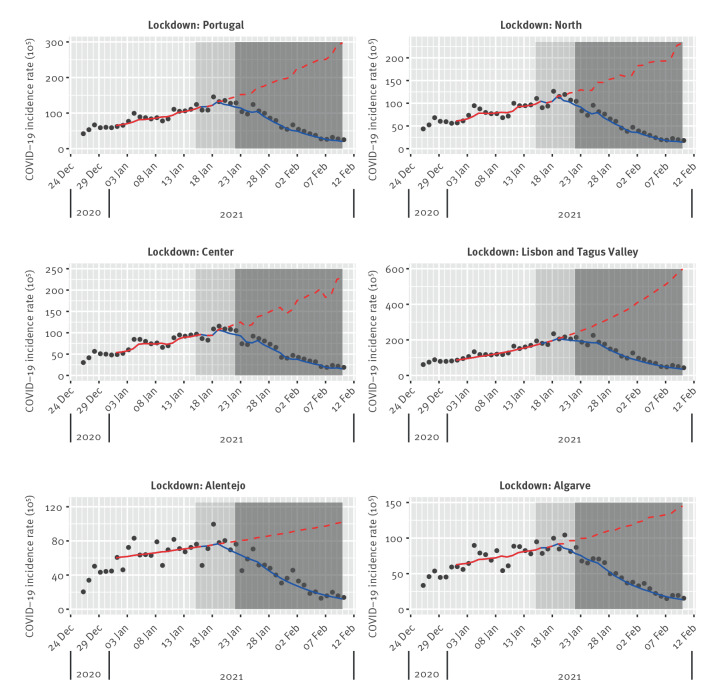
COVID-19 daily incidence rates^a^ during the third epidemic wave, across Portugal and five regional health administrations, 26 December 2020–10 February 2021 (n = 372,680)

In the pre-lockdown study period (26 December 2020–14 January 2021) after the third wave onset, the overall COVID-19 incidence rate increased on average by 3.8% per day (IRR: 1.038; 95% CI: 1.029–1.046). Significant increasing trends were observed in all regions, being highest in Lisbon and Tagus Valley (4.9% per day; IRR: 1.049; 95% CI: 1.041–1.058) (Table 3 and Figure 3).

**Table 3:** Daily trends in COVID-19 incidence rate before and after lockdown implementation with and without school closure, across Portugal and in five regional health administrations, 26 December 2020–10 February 2021 (n = 372,680)

Area	Study period						Change in trend					
	Pre-lockdown 26 December 2020–14 January 2021		Lockdown without school closure 15–21 January 2021		Lockdown with school closure 22 January–10 February 2021		Without school closure			With school closure		
	IRR	95% CI	IRR	95% CI	IRR	95% CI	Change in IRR	95% CI	p value^a^	Change in IRR	95% CI	p value^a^
Portugal	1.038	1.029–1.046	0.966	0.935–0.998	0.897	0.846–0.951	0.931	0.909–0.954	< 0.001	0.928	0.904–0.953	< 0.001
**Regional health administration**												
North	1.032	1.023–1.042	0.947	0.914–0.981	0.892	0.836–0.951	0.917	0.893–0.942	< 0.001	0.942	0.915–0.969	< 0.001
Center	1.035	1.025–1.044	0.960	0.926–0.996	0.895	0.837–0.957	0.928	0.903–0.954	< 0.001	0.932	0.904–0.960	< 0.001
Lisbon and Tagus Valley	1.049	1.041–1.058	0.982	0.953–1.013	0.896	0.847–0.948	0.936	0.915–0.958	< 0.001	0.912	0.890–0.935	< 0.001
Alentejo	1.013	1.000–1.026	0.955	0.907–1.006	0.903	0.822–0.993	0.942	0.907–0.980	0.003	0.946	0.906–0.987	0.011
Algarve	1.021	1.012–1.030	0.953	0.918–0.989	0.899	0.839–0.964	0.934	0.908–0.960	< 0.001	0.944	0.914–0.974	< 0.001

COVID-19: coronavirus disease; CI: confidence interval; IRR: incidence rate ratio.

^a^ Two-sided Wald test p values were obtained from negative binomial regression models. A p value < 0.05 was considered evidence of statistical significance.

COVID-19: coronavirus disease.

^a^ Per 100,000 population.

The shaded light grey area represents the period after lockdown implementation without school closure. The shaded dark grey area represents the period after lockdown implementation with school closure. Red and blue solid lines represent the fitted incidence rates before and after lockdown, respectively. The red dashed line represents the predicted incidence rate in a contrafactual scenario, i.e. if the lockdown had not been implemented.

A statistically significant change in trend was observed for the overall incidence rate and all regions during the lockdown study period without school closure (15–21 January 2021). Significant declining trends were observed across mainland Portugal (−3.4% per day; IRR: 0.966; 95% CI: 0.935–0.998) and all regions except Lisbon and Tagus Valley and Alentejo. In the lockdown with school closure study period (22 January–10 February 2021), a statistically significant change in trend was observed for the overall incidence rate and all regions. Significant declining trends were observed in mainland Portugal (−10.3% per day; IRR: 0.897; 95% CI: 0.846–0.951) and all regions. Declining trends ranged from –10.8% (IRR: 0.892; 95% CI: 0.836–0.951) in North and −9.7% (IRR: 0.903; 95% CI: 0.822–0.993) in Alentejo (Table 3 and Figure 3).

The absolute effects on the subsequent 14-day period associated with tiered NPIs and lockdown (9–22 November 2020 for tiered NPIs and 15–28 January 2021 for lockdown) yielded 137 (19.0%) cases and 437 (27.7%) cases per 100,000 population potentially averted, respectively (Table 4).The biggest absolute effect estimated occurred in the North for tiered NPIs (317 potential cases (25.7%) averted per 100,000 population) and for Lisbon and Tagus Valley in lockdown (704 potential cases (26.9%) averted per 100,000 population).

**Table 4:** COVID-19 cumulative incidence rates^a^ for tiered non-pharmaceutical interventions and lockdown, across Portugal and in five regional health administrations, 9–22 November 2020 (tiered non-pharmaceutical interventions) (n = 74,125) and 15–28 January 2021 (lockdown) (n = 162,329)

Area	Cumulative incidence observed with restrictive measures^a^	Cumulative incidence predicted without restrictive measures^a^	95% CI	Potential cumulative incidence averted^a^	95% CI	
**Tiered NPIs: 14-day observation period during 9–22 Nov 2020**						
Portugal	720	857	802 to 916	137		82 to 196
**Regional health administration**						
North	1,233	1,550	1,454 to 1,651	317		222 to 419
Center	389	460	427 to 495	71		39 to 107
Lisbon and Tagus Valley	631	705	663 to 751	74		31 to 120
Alentejo	230	226	205 to 250	−4		−25 to 19
Algarve	273	318	281 to 359	45		9 to 87
**Lockdown: 14-day observation period during 15–28 Jan 2021**						
Portugal	1,577	2,013	1,808 to 2,245	437		231 to 668
**Regional health administration**						
North	1,302	1,715	1,526 to 1,930	413		224 to 628
Center	1,249	1,615	1,429 to 1,827	366		181 to 578
Lisbon and Tagus Valley	2,615	3,319	2,992 to 3,685	704		377 to 1070
Alentejo	907	1,105	934 to 1,311	199		27 to 404
Algarve	1,078	1,346	1,193 to 1,519	267		115 to 440

COVID-19: coronavirus disease; NPIs: non-pharmaceutical interventions.

^a^ Per 100,000 population.

The models showed acceptable goodness-of-fit with Pearson’s Chi-squared tests all indicating insufficient evidence of lack of fit (p > 0.05). Graphical depictions of residuals from regression models demonstrated no quantile deviations detected, supporting the specification of a negative binomial model to estimate trends in COVID-19 cases.

### Control for confounding

After adjusting for average temperature, a potential confounding variable that could affect the number of COVID-19 cases, our analyses suggest that tiered NPIs are still associated with COVID-19 incidence trends. Furthermore, trends found with adjusted regression models (Table 5) did not differ significantly from non-adjusted regression models (Table 2).

**Table 5:** Daily trends in COVID-19 incidence rate adjusted for temperature before and after implementation of tiered non-pharmaceutical interventions, across Portugal and in five regional health administrations, 30 September–18 December 2020 (n= 297,754)

Area	Study period				Change in trend	95% CI	p value^a^
	Pre-tiered NPIs 30 Sep–8 Nov 2020		Tiered NPIs 9 Nov–18 Dec 2020				
	IRR	95% CI	IRR	95% CI			
Portugal	1.028	1.025–1.031	0.980	0.972–0.988	0.954	0.949–0.959	< 0.001
**Regional health administration**							
North	1.032	1.030–1.035	0.968	0.961–0.976	0.938	0.933–0.943	< 0.001
Center	1.038	1.035–1.041	0.991	0.982–1.000	0.955	0.949–0.961	< 0.001
Lisbon and Tagus Valley	1.019	1.016–1.022	0.990	0.982–0.998	0.972	0.967–0.976	< 0.001
Alentejo	1.021	1.017–1.025	1.015	1.003–1.027	0.994	0.986–1.001	0.112
Algarve	1.029	1.023–1.035	0.986	0.971–1.002	0.959	0.949–0.968	< 0.001

COVID-19: coronavirus disease; CI: confidence interval; IRR: incidence rate ratio; NPIs: non-pharmaceutical interventions.

^a^ Two-sided Wald test p values were obtained from negative binomial regression models. A p value < 0.05 was considered evidence of statistical significance.

Our analyses suggest that lockdown implementation is associated with COVID-19 incidence trends after adjusting for temperature in the overall sample. Trends found with adjusted regression models (Table 6) differed significantly from non-adjusted regression models (Table 3) for North, Alentejo and Algarve regions. However, our estimates for the adjusted regression models show that, for all regions, lockdown with school closure has a significant effect in reverting the increasing COVID-19 incidence trend.

**Table 6:** Daily trends in COVID-19 incidence rate adjusted for temperature before and after lockdown implementation with and without school closure, across Portugal and in five regional health administrations, 26 December 2020–10 February 2021 (n = 372,680)

Area	Study period						Change in trend					
	Pre-lockdown 26 December 2020–14 January 2021		Lockdown without school closure 15–21 January 2021		Lockdown with school closure 22 January–10 February 2021		Without school closure			With school closure		
	IRR	95% CI	IRR	95% CI	IRR	95% CI	Change in IRR	95% CI	p value^a^	Change in IRR	95% CI	p value^a^
Portugal	1.038	1.029–1.047	0.963	0.916–1.012	0.897	0.821–0.980	0.927	0.890–0.966	< 0.001	0.931	0.896–0.969	< 0.001
**Regional health administration**												
North	1.033	1.024–1.043	0.938	0.890–0.988	0.892	0.812–0.980	0.907	0.870–0.947	< 0.001	0.951	0.912–0.992	0.020
Center	1.035	1.025–1.045	0.958	0.910–1.009	0.895	0.816–0.981	0.926	0.888–0.966	< 0.001	0.934	0.897–0.972	< 0.001
Lisbon and Tagus Valley	1.049	1.041–1.058	0.983	0.944–1.024	0.896	0.834–0.963	0.937	0.907–0.968	< 0.001	0.911	0.883–0.940	< 0.001
Alentejo	1.012	0.998–1.026	0.964	0.890–1.044	0.903	0.782–1.043	0.953	0.892–1.017	0.146	0.937	0.878–0.999	0.046
Algarve	1.020	1.009–1.031	0.961	0.900–1.027	0.899	0.797–1.014	0.943	0.892–0.997	0.038	0.935	0.886–0.987	0.015

COVID-19: coronavirus disease; CI: confidence interval; IRR: incidence rate ratio.

^a^ Two-sided Wald test p values were obtained from negative binomial regression models. A p value < 0.05 was considered evidence of statistical significance.

### Sensitivity analysis

Point estimates varied between −1.8% and −2.0% for the declining trend associated with tiered NPIs, across the lag period (considering a median incubation period between 2 and 12 days).

Trends associated with the lockdown without school closure, ranged from a 1.2% increase and a −12.6% decrease in the incidence rate per day, in Portugal, as the lag period varied. Trends associated with the school closure in Portugal varied less across the lag period, ranging between −9.7% and −10.5% (Supplementary Table S1: Sensitivity analysis for tiered NPIs and Table S2: Sensitivity analysis for lockdown).

## Discussion

Tiered NPIs were implemented in Portugal in the middle of the second epidemic wave. Our findings indicate that, while the restrictions had an effect in reverting the COVID-19 incidence trend, this effect resulted in only a modest declining trend. In addition, the highest declining trend was observed in the North region, which had strict NPIs for most municipalities over a long duration. As these restrictions limited movement especially on weekends, this suggests that a significant reduction of COVID-19 incidence is achieved through a combination of the number of municipalities under lockdown and time under stringent NPIs.

Our results concerning the effect of tiered NPIs in mitigating the spread of COVID-19 are in line with a study which simulated the effects of regional restrictive measures in Italian regions, adopted according to the saturation of their hospital capacity [7]. The study confirmed the effectiveness of regional lockdowns in avoiding future waves of the epidemic. However, we stress that this measure was proven to be beneficial in mitigating possible outbreaks rather than suppressing the epidemic. Similar results were found in a modelling study from the United Kingdom which projected a reduction of 48,600 COVID-19 deaths and 238,000 hospital admissions with tiered NPIs implemented from October 2020 to March 2021 [20]. Additionally, a modelling study on the effectiveness of a four-tier response among 19 cities/regions in mainland China concluded that later implementation of lockdowns and physical distancing measures may result in three to ten times higher peak of infections, revealing that stringent NPIs are necessary for achieving a significant reduction in COVID-19 incidence in cities with higher epidemic risk [21].

Our findings indicate that a nationwide lockdown without school closure was effective in reducing COVID-19 cases in Portugal. This result is in line with findings from a modelling study which estimated a reduction of 35% in the COVID-19 effective reproduction number for a lockdown in Northern Ireland with schools closed, compared to a 2–10% reduction for less stringent NPIs applied in England during the same period of time [20]. For Lisbon and Tagus Valley and Alentejo regions, our results suggest that a lockdown without school closure was successful in changing the COVID-19 incidence trend (mitigation effect), although the declining effect was not significant. One possible explanation is related to the highly transmissible SARS-CoV-2 Alpha variant (B.1.1.7) which was progressively increasing its frequency in Portugal from the beginning of January 2021 [22]. Moreover, data estimated that this lineage was more prevalent in the Lisbon and Tagus Valley region, representing 50% of the new COVID-19 cases by the end of January [23].

Our results suggest that, in Portugal, school closure achieved the strongest and most rapid effect to reduce COVID-19 incidence in the third epidemic wave, with a certain degree of homogeneity across all mainland regions. Our findings are consistent with a study which found that school closure in the United States was associated with decreased COVID-19 incidence and states that closed schools earlier had the largest relative reduction in COVID-19 incidence [24]. Similar results were found in a study assessing the independent effects of COVID-19 restrictions across 175 countries, estimating a larger reduction in COVID-19 incidence for school and workplace closure [6]. Furthermore, the population of children and youth (aged ≤ 19 years) in Portugal showed a seroprevalence close to that of the adult population, suggesting that they did not have a lower infection rate; therefore, decreased COVID-19 incidence observed after school closure is consistent with spread disruption among these age groups [25]. Nevertheless, the association of school closure with a decline in COVID-19 cases has several potential explanations besides a direct causal effect of transmission control among children. School closure causes societal changes in family routines, with parents resorting to alternative childcare options, modifying work schedules, decreasing mobility and increasing telework [24]. In fact, a study on the effects of school closure and telework, conducted in France, shows that school closure alone would have limited benefit in reducing the peak incidence but when coupled with 25% adults teleworking, an 8-week school closure would be enough to delay the peak by almost 2 months with an ca 40% reduction of the case incidence [26]. In Portugal, mobility trends for places of work decreased ca 10% after school closure implementation (compared to the period of lockdown without school closure (15–21 January 2021)), hence, suggesting an altered adult behaviour that may have played an important part in decreasing COVID-19 spread [14]. 

In sensitivity analyses, trends associated with tiered NPIs across different lag periods were similar to trends found with the primary analysis. Trends associated with the lockdown without school closure varied for shorter and longer lagging times. A possible explanation is the insufficient time to account for an effect of the lockdown implementation in shorter lags (especially, in face of the high COVID-19 incidence observed at that period) and because of the overlapping effect of the school closure implementation for longer lags. Trends associated with school closure implementation across different lag periods were similar to trends found with the primary analysis, demonstrating the robustness of the model.

Our study has some limitations. Temperature was introduced in our regression models to control for potential meteorological confounding; similar adjustment was performed in other studies and may be considered a strength of this study [27]. Nonetheless, it is not possible to fully isolate potential effects of restrictive measures from weather drivers in reducing COVID-19 incidence as, for example, we did not account for effects of humidity in our regression models. We point out that, although we expect SARS-CoV-2 to show environmental sensitivity, experimental evidence suggests a limited effect of these variables in reducing disease transmission [28]. Another limitation of our study is the use of average temperature, which may not capture a large spread in climatic conditions. However, Portugal does not have a large spread in latitude and, therefore, this effect is not expected to substantially influence our results. Importantly, in the presence of controls (temperature), no significant changes in trends for the COVID-19 incidence in Portugal associated with tiered NPIs and lockdown were found. Such results demonstrate a robust impact of the restrictive measures adopted on daily new cases of COVID-19, that cannot be attributed to temperature patterns. Moreover, similar studies show that temperature is not the dominant factor in SARS-CoV-2 spread; a study on temperature dependence of SARS-CoV-2 transmission found that virus spread is slower at high temperature, however temperature only explains 18% of the variability of the disease spread [29]. Another limitation is related to estimates of daily COVID-19 incidence rates used in our analysis, derived from the estimated number of daily COVID-19 cases by date of symptom onset. Such estimation was based upon observed cases or, when missing, imputed cases by date of symptom onset. However, our imputation method includes a missing at random assumption, which in this case, implies that the time between disease onset and reporting is the same for individuals with and without available symptom onset date. This could be inaccurate if there are many asymptomatic and presymptomatic cases among the reported COVID-19 cases. Surveillance data used in this study do not include information on cases’ symptomatology. Nevertheless, related literature using imputation of disease onset in order to estimate the COVID-19 epidemic curve indicates that results are relatively stable regardless of whether the presence of asymptomatic and presymptomatic cases is accounted for [30]. Finally, we did not take into account the demographic and socioeconomic structure of the population in each region in our analysis. Older age groups are more likely to comply with strict NPIs, and therefore, age structure may be a source of bias when comparing the stringent NPIs impact across regions [31]. Also, sociodemographic characteristics, such as sex and socioeconomic status, have also been associated with compliance [31]. Nonetheless, while it is fair to say that demographic and socioeconomic factors can influence adherence to stringent NPIs, we expect this effect to be low as lockdowns across Portugal were placed into law through legislation and, therefore, provided tools for policy implementation backed by enforcement.

## Conclusion

Our results indicate a likely effect of tiered NPIs in reverting a rise in COVID-19 incidence; however, this effect resulted in only a modest declining trend. A lockdown without school closure seems to be effective in slowing transmission, but the addition of school closure is likely to provide the strongest effect in reducing COVID-19 incidence, thus reducing pressure on health services. Results from these findings presented herein emphasise the importance of early and assertive decision-making to contain the pandemic.

## Supplementary Data

447000 Supplement
