# Supplementary material for: Impact of stringent non-pharmaceutical interventions applied during the second and third COVID-19 epidemic waves in Portugal, 9 November 2020 to 10 February 2021: an ecological study
Source: Euro Surveill. 2022 Jun 9;27(23):2100497. doi: 10.2807/1560-7917.ES.2022.27.23.2100497 (PMC9198658; doi:10.2807/1560-7917.ES.2022.27.23.2100497)
Supplement: Supplement [file 21-00497_TORRES_Supplement.pdf]

SUPPLEMENTARY MATERIAL

This supplementary material is hosted by Eurosurveillance as supporting information alongside the article "Impact of strict social distancing measures applied in Portugal during the second and third COVID-19 epidemic waves", on behalf of the authors, who remain responsible for the accuracy and appropriateness of the content. The same standards for ethics, copyright, attributions and permissions as for the article apply. Supplements are not edited by Eurosurveillance and the journal is not responsible for the maintenance of any links or email addresses provided therein.

Figure S1. (1) COVID-19 incidence rates[a] before and after tiered non-pharmaceutical interventions and lockdown, Portugal, 30 September 2020–10 February 2021 (2) Average daily temperatures, Portugal, 30 September 2020–10 February 2021

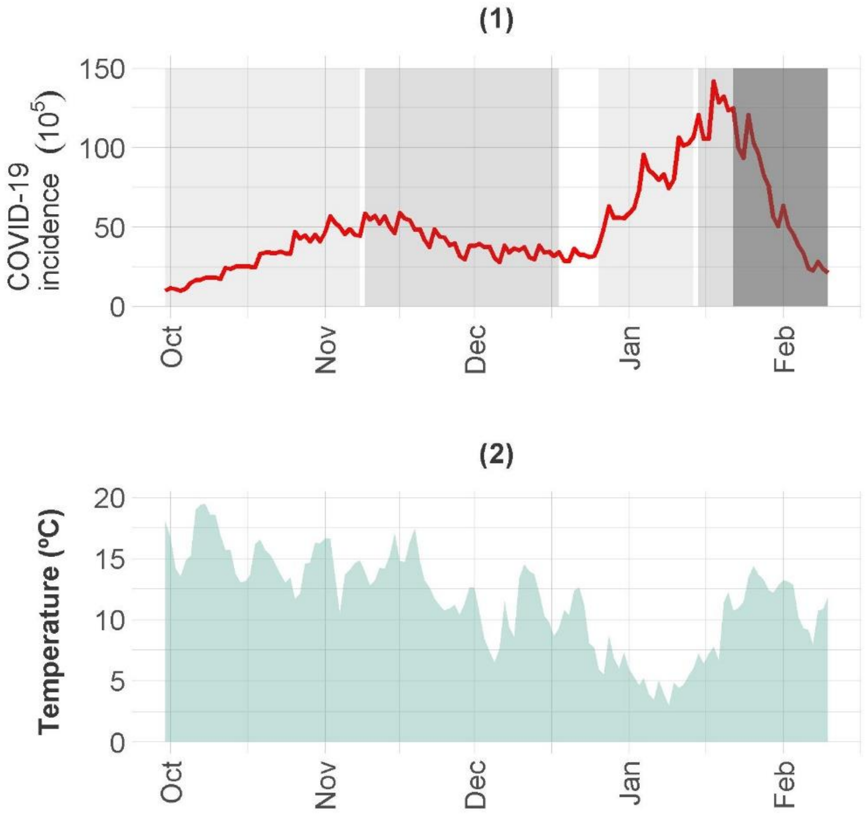

COVID-19: coronavirus disease; NPI: non-pharmaceutical interventions.

a Per 100,000 population.

The lighter shaded grey areas represent the analysis periods before tiered non-pharmaceutical interventions and lockdown. The medium and darker shaded grey areas represent the analysis periods after the implementation of tiered non-pharmaceutical interventions and lockdown.

Table S1. Sensitivity analyses with varying time lag for daily trends in COVID-19 incidence rate before and after implementation of tiered non-pharmaceutical interventions, Portugal, 30 September–18 December 2020 (n= 297,754)

Lag	Study period				Change in trend	95% CI	p value[a]
	Pre-tiered NPIs 30 Sep–8 Nov 2020		Tiered NPIs 9 Nov–18 Dec 2020				
	IRR	95% CI	IRR	95% CI			
2	1.035	1.032-1.037	0.982	0.975-0.990	0.949	0.945-0.954	<0.001
3	1.032	1.029-1.035	0.982	0.974-0.989	0.951	0.947-0.956	<0.001
4	1.030	1.027-1.033	0.981	0.974-0.989	0.953	0.948-0.958	<0.001
5	1.028	1.025-1.031	0.981	0.973-0.989	0.954	0.949-0.959	<0.001
6	1.026	1.023-1.029	0.981	0.972-0.989	0.955	0.950-0.961	<0.001
7	1.024	1.021-1.027	0.980	0.972-0.989	0.957	0.951-0.963	<0.001
8	1.022	1.019-1.025	0.980	0.971-0.989	0.959	0.953-0.964	<0.001
9	1.020	1.017-1.023	0.980	0.971-0.989	0.961	0.955-0.967	<0.001
10	1.017	1.014-1.020	0.980	0.971-0.989	0.963	0.957-0.969	<0.001
11	1.016	1.012-1.019	0.980	0.971-0.989	0.965	0.959-0.971	<0.001
12	1.014	1.010-1.017	0.980	0.970-0.990	0.967	0.960-0.974	<0.001

COVID-19: coronavirus disease; CI: confidence interval; IRR: incidence rate ratio; NPIs: non-pharmaceutical interventions.

[a] Two-sided Wald test p values were obtained from negative binomial regression models. A p value < 0.05 was considered evidence of statistical significance.

Table S2. Sensitivity analyses with varying time lag for daily trends in COVID-19 incidence rate before and after lockdown implementation with and without school closure, Portugal, 26 December 2020–10 February 2021 (n = 372,680)

Lag	Study period						Change in trend					
	Pre-lockdown 26 December 2020–14 January 2021		Lockdown without school closure 15–21 January 2021		Lockdown with school closure 22 January–10 February 2021		Without school closure			With school closure		
	IRR	95% CI	IRR	95% CI	IRR	95% CI	Change in IRR	95% CI	p value[a]	Change in IRR	95% CI	p value[a]
2	1.042	1.033-1.051	1.012	0.979-1.047	0.903	0.852-0.958	0.971	0.948-0.996	0.023	0.892	0.870-0.915	<0.001
3	1.042	1.033-1.051	0.996	0.963-1.030	0.901	0.849-0.956	0.956	0.932-0.980	<0.001	0.904	0.881-0.928	<0.001
4	1.041	1.032-1.049	0.981	0.950-1.013	0.899	0.848-0.952	0.942	0.920-0.965	<0.001	0.916	0.893-0.940	<0.001
5	1.038	1.029-1.046	0.966	0.935-0.998	0.897	0.846-0.951	0.931	0.909-0.954	<0.001	0.928	0.904-0.953	<0.001
6	1.034	1.026-1.043	0.952	0.921-0.983	0.896	0.843-0.951	0.920	0.898-0.943	<0.001	0.941	0.916-0.967	<0.001
7	1.031	1.022-1.039	0.938	0.907-0.969	0.895	0.841-0.953	0.910	0.887-0.933	<0.001	0.955	0.927-0.983	0.002
8	1.028	1.019-1.036	0.922	0.891-0.955	0.896	0.838-0.957	0.898	0.874-0.922	<0.001	0.971	0.941-1.002	0.068
9	1.028	1.019-1.037	0.905	0.874-0.938	0.898	0.838-0.962	0.881	0.857-0.904	<0.001	0.992	0.959-1.026	0.637
10	1.025	1.016-1.034	0.892	0.859-0.925	0.899	0.835-0.969	0.870	0.846-0.895	<0.001	1.008	0.971-1.047	0.668
11	1.019	1.010-1.028	0.883	0.851-0.917	0.899	0.832-0.972	0.866	0.842-0.891	<0.001	1.018	0.978-1.060	0.379
12	1.012	1.003-1.021	0.874	0.840-0.909	0.902	0.828-0.982	0.864	0.838-0.890	<0.001	1.032	0.986-1.081	0.176

COVID-19: coronavirus disease; CI: confidence interval; IRR: incidence rate ratio.

[a] Two-sided Wald test p values were obtained from negative binomial regression models. A p value < 0.05 was considered evidence of statistical significance.
